# Planning dietary improvements without additional costs for low-income individuals in Brazil: linear programming optimization as a tool for public policy in nutrition and health

**DOI:** 10.1186/s12937-019-0466-y

**Published:** 2019-07-20

**Authors:** Eliseu Verly-Jr, Rosely Sichieri, Nicole Darmon, Matthieu Maillot, Flavia Mori Sarti

**Affiliations:** 1 Department of Epidemiology, Institute of Social Medicine, Rio de Janeiro State University, Rua São Francisco Xavier 524, Rio de Janeiro, 20550-013 Brazil; 2 MOISA, INRA, CIHEAM-IAMM, CIRAD, Montpellier SupAgro, Université de Montpellier, 34060 Montpellier, Cedex 2 France; 3 MS-Nutrition, Faculté de Médecine La Timone, 27, bd Jean Moulin, 13385 Marseille, France; 4 Center for Research in Complex Systems Modeling, School of Arts, Sciences and Humanities, University of São Paulo, Av. Arlindo Bettio, 1000, São Paulo, 03828-000 Brazil

**Keywords:** Linear programming, Diet cost, Nutrient adequacy, Food planning, Diet modeling

## Abstract

**Background:**

Meeting nutrient intake recommendations may demand substantial modifications in dietary patterns, and may increase diet cost. Incentives for modifying one’s dietary intake that disregard prices are unlikely to be effective in the general population, especially among low-income strata, due to the high percentage of income committed to food purchases. The aim of this study is to evaluate how much the nutrient content can be increased through a modeled diet, without any cost increase, for low-income Brazilian households.

**Methods:**

Low-income households were selected from the Household Budget Survey (24,688 households) and National Dietary Survey (6,032 households, 16,962 individuals), from where we obtained food prices and consumption data. Food quantities were modeled using linear programming to find diets that meet nutritional recommendations in two sets of models: cost-constrained (the cost should not be higher than the observed diet cost) and cost-free. Minimum and maximum amounts of each food in the modelled diets were allowed at three levels of food acceptability: rigorous (least deviance from the current observed diets), moderate, and flexible (higher deviance from the current observed diets).

**Results:**

We found no feasible solution that would accommodate all the nutritional targets. The most frequent limiting nutrients were calcium; vitamins D, E, and A; zinc; fiber; sodium; and saturated and trans-fats. However, increases in nutrient contents were observed, especially for fiber, calcium, copper, magnesium, vitamin A, vitamin C, and vitamin E. In general, the best achievement was obtained with cost-free models. Fruits and beans increased in all models; large increase in whole cereals was observed only in the flexible models; large increase in vegetables was observed only in the cost-free models; and fish increased only in the cost-free models. Reductions were observed for rice, red and processed meats, sugar-sweetened beverages, and sweets. The mean observed cost was US$2.16 per person/day. The mean cost in the cost-free models was US$2.90 (moderate), US$2.70 (rigorous), and US$2.60 (flexible).

**Conclusion:**

The complete nutritional adequacy is unattainable, although feasible changes would substantially improve diet quality by improving nutrient content without additional costs.

## Background

A high prevalence of inadequate nutrient intakes is still observed in developed [1, 2] and developing countries. In Brazil, the prevalence of inadequacy higher than 70% was described for calcium, vitamin A, vitamin D, vitamin E, and magnesium; in addition, inadequacy ranged from 30 to 50% for vitamins C, B1, B6, and riboflavin in adults [3] and elderly population [4]. Improvements in dietary quality and the reduction of intake inadequacy may require substantial modifications in dietary patterns. However, the adoption of nutrient-based recommendations implies that they are affordable and culturally acceptable [5, 6].

Concerning dietary costs, studies conducted in developed and developing countries, including Brazil [7], have shown that families in low socioeconomic strata consume lower amounts of healthy foods (such as whole cereals, fish, dairy, fruits and vegetables) in comparison with families in higher socioeconomic strata [8]. Financial constraints may orient people toward choosing dietary patterns that have low micronutrient density [9] and high energy density [10]. Foods with higher micronutrient content usually have higher prices per calorie than processed foods or energy-dense foods [11]. Incentives for modification of dietary intake that disregard prices are unlikely to be effective among the population, especially individuals in lower socioeconomic strata, due to the high percentage of income committed to food purchases. Evidence from Australia has shown that among families with relatively low income, approximately one-third of the household budget should be used for food purchases to fulfill nutrient-based recommendations [12]. In Ireland, the percentage varied from 40% in households of a single elderly person to 80% in households having a single parent with two children [13]. In Brazil, nearly half of the population earns an income up to one minimum wage, which was estimated to be insufficient to meet official food-based dietary guidelines [14].

Considering food prices is a necessity when promoting healthy diets among populations worldwide. Linear programming is a useful methodology to identify the combination of foods that can achieve nutritional recommendations at the lowest cost. The method was proposed as early as 1959 [15]; however, only recently have its applications been directed towards the development of more realistic dietary guidelines [16] and the identification of more sustainable food choices [17]. In particular, mathematical minimization of the distance between observed diets and optimized diets allows encompassing simultaneously nutrition, affordability and local food preferences in the same models [18–20].

The aim of this study is to evaluate how much the nutrient content can be increased through a modeled realistic diet, without any cost increase, for low-income Brazilian households. Additionally, we compared the cost-constrained versus cost-free optimized diets to assess the effect of budgets on food selection and nutritional content.

## Materials and methods

### Dietary data

We used data from the National Dietary Survey (NDS), a subsample of the Household Budget Survey (HBS), both held between March 2008 and March 2009 by the Brazilian Institute of Geography and Statistics. The HBS collected information on food purchases for a 1-week period from 55,970 households, among which 13,460 households were also randomly selected to participate in the NDS. In the NDS, all individuals aged 10 years or older filled in two non-consecutive food records during the time-frame of the HBS, with a 97% response rate for the second food record. A two-stage sampling process was adopted: in the first stage, census tracts were randomly selected; in the second stage, households were randomly selected within census tracts. Census tracts (*n* = 1,280) were grouped into 550 strata with geographical and socioeconomic homogeneity, and the number of tracts in each stratum was proportional to the number of households in the stratum. Data collection procedures are described elsewhere [21].

### Determination of the mean observed diet

Brazil is a large country with differences in culture and economy throughout the states. Then, these analyses were intended to be performed for each stratum rather than the overall population, to account for local dietary patterns and food prices. However, to achieve a higher precision regarding the mean food consumption and price, sampling strata were collapsed into four higher geographic areas: capital of the state, metropolitan area, rest of the state, and rural area. This was done for each of the 26 Brazilian states and one Federal District, but in 18 states there were no strata for the metropolitan region, thus they were collapsed into three instead of four geographic areas, totaling 89 aggregated strata, called geographic strata (GS). Thus, the unit of analysis is the GS, and the model’s input (constraints and decision variables) and output (optimized nutrient and food contents) are GS-specific. For the analyses, we selected only households with a monthly per-capita income less than or equal to one minimum wage (Brazilian Reals BRL 415.00 equivalent to US$ 179.65 at January 30th, 2009). The final sample was 24,688 households from a total of 55,970 in the full HBS sample, and 6,032 households (16,962 individuals) in the NDS subsample.

Mean food intakes were calculated for each GS using dietary information from the two food records for each individual, when available, weighted by sampling weights. Dietary data for pregnant and breastfeeding women (*n* = 1254) were not considered. Overall mean food intake – that is, the mean intake over all 89 GS – is here referred to as “mean observed diet”. A food composition dataset compiled for the Brazilian nutritional survey [22] was used in this study. The nutrient composition of foods clustered into food items (e.g. different types of rice into “rice”) was estimated as the mean composition of the food included in the subgroup, weighted by frequency of reporting in the dietary survey. NDS participants reported 305 food items, mostly clustered into 110 food items (e.g., different types of cakes were all categorized simply as “cakes”). We excluded non-food nutrient and energy sources from the food list, such as coffee and tea (without sugar) and alcoholic beverages. The resulting list contained 105 foods for the overall sample, varying from 37 to 92 items depending on the GS. All food items were categorized into six food groups: fruits and vegetables; seeds and legumes; cereals; dairy; meats and eggs; oils; and other foods, and 26 food subgroups.

### Food-price data

We extracted the food prices from the HBS database. Each household member had registered the amount and price of each item purchased in a 1-week period, and the mean price for each food item was calculated for each GS. These prices were matched to their counterparts in the food consumption dataset, according to the GS; thus, the price variations across the sampled areas were preserved. For aggregated food items (that is, food items comprising different variations of a given food), we used the mean price weighted by the frequency of reporting of each food, stratified by GS. Prices of the foods as purchased were converted into prices per 100 g of edible portion using proper correction and cooking factors.

Household visits in each stratum were uniformly distributed throughout the 12-month period to encompass seasonal variations in both food intakes and prices. Considering the variation in food prices during the period of data collection, all prices were deflated to the same reference date (January 2009) using official inflation rates (National Consumers’ Prices Index).

### Linear programming models

An optimization model is defined based on an objective function that depends on diverse variables (i.e., decision variables) and restricted by various constraints [17]. In this study, the constraints imposed in the models were energy and nutrients, food quantities, and total cost of the diet. Decision variables were the amount (in grams) of food items included in each GS model. Food items not reported by any individual in a given GS were allowed to be introduced since they were reported by individuals from another GS within the same state. We performed six sets of optimization models: cost-free and cost-constrained, each with three levels of food acceptability. Food acceptability referred to the maximum theoretically acceptable changes in food quantities, compared with the current observed diet. For each set, 89 optimization models were run, one for each GS. The models accounted for food price and consumption, obtained at GS level. Thus, the total number of optimization models was 534 (6 × 89). All of the models were aimed at finding combinations of food items and their quantities while respecting nutritional and acceptability constraints, while minimizing the departure from the current observed diet in each one of those 89 GS. Cost constraining was also applied in the cost-constrained models.

The modeling process was conducted in two steps. In step one, for each GS we ran a model that included all the nutritional constraints. In case of model unfeasibility, that is, one or more nutritional constraints could not be attained in a given GS, a built-in algorithm in the PROC OPTMODEL (SAS software) was used to identify the nutritional constraints that caused unfeasibility (referred below as “limiting nutrients”). The occurrence of model unfeasibility led to step two, in which constraints on limiting nutrients were removed and an additional term was added in the objective function, in addition to the food departure minimization. This minimized the undesirable deviations from the nutritional targets for the limiting nutrients. With this term, the models allowed a less stringent level of nutritional exigency. That is, the best possible achievement of nutritional targets was expected rather than strict adherence to the recommended levels for each nutrient.

### Nutritional constraints

The whole set of nutritional constraints is presented in Table 1. It includes macronutrients, saturated fatty acids, fibers, trans-fats, and added sugars. For calcium; magnesium; iron; phosphorus; copper; zinc; vitamins A, B1, B2, B6, B12, C, D, and E; niacin; and folate, the cutoff points were derived from the Recommended Dietary Allowances (RDA) issued by the US Institute of Medicine [23, 24]. The RDA refers to the intake level estimated to achieve the nutritional requirements of 97.5% of healthy individuals in each age and sex group [24]. The constraint for each nutrient in each of the 534 models corresponded to the mean recommended values (i.e., mean of the RDA values over age-sex groups) weighted by the frequency of age-sex group in the whole population. Nutrients without an RDA were not constrained in the models. Due to absence of information on the accuracy of estimates for added salt in food preparations, the sodium content in optimized diets was constrained to be no higher than the content in the observed diet for each GS. Red and processed meat was constrained to a maximum of 500 g/week [25]. Total energy content was constrained to be equal to the mean caloric intake in the corresponding GS. The optimized content for healthy and beneficial nutrients was not allowed to be lower than the current mean content for each GS. Similarly, optimized content was not allowed to be higher than the current mean for nutrients whose intake should be limited, namely sodium, saturated and trans-fats, and added sugar.

**Table 1:** Nutritional constraints imposed on the linear programming models

Nutrient	Unit	Constraints
Energy	Kcal	= observed^a^
Carbohydrates ^b^	%kcal	45–55
Free sugar ^c^	%kcal	< 10
Total fiber ^b^	g	≥31
Protein ^b^	%kcal	≥10
Total fat ^b^	%kcal	25–35
Saturated fat ^c^	%kcal	< 10
Trans fat ^c^	%kcal	< 1
Sodium	mg	≤ observed^a^
Calcium ^d^	mg	≥1,021
Copper ^d^	mg	≥1.1
Iron ^d^	mg	≥10.7
Phosphorus ^d^	mg	≥888
Magnesium ^d^	mg	≥377
Zinc ^d^	mg	≥11.7
Niacin ^d^	mg	≥16.4
Vitamin A ^d,e^	mcg	≥803
Thiamin ^d^	mg	≥1.3
Riboflavin ^d^	mg	≥1.5
Vitamin B6 ^d^	mg	≥1.6
Vitamin B12 ^d^	mcg	≥4.2
Vitamin C ^d^	mg	≥84
Vitamin D ^e^	mcg	≥10
Vitamin E ^d^	mg	≥12
Folate ^d,f^	mcg	≥426

^a^ Mean observed caloric (and sodium) intake for the corresponding GS

^b^ Institute of Medicine (2001) [24]

^c^ World Health Organization (2003) [31]

^d^ Derived from the Recommended Dietary Allowance

^e^ Micrograms of Retinol Equivalent Activity

^f^ Micrograms of Dietary Folate Equivalent

### Cost constraint

In the cost-free models, no cost constraint was imposed. In the cost-constrained models, total cost of the optimized diet was set to be equal to the current cost. The current cost of a diet was obtained for each GS by summing the mean price and multiplying it by the mean amount of each food item. Cost of the mean observed diet (i.e., mean intake across all 89 GS) is referred to as “mean observed cost”.

### Acceptability constraints

Acceptability constraints are boundaries in which optimized food items may deviate from observed mean intakes, avoiding culturally or socially unacceptable optimized diets. Boundaries were calculated for each food item, among the whole population (not only low-income individuals) as follows. First, mean food intakes within each stratum (from all 550 strata in the full sample) were obtained. To account for food preferences across the country, we obtained the acceptability constraints for each region (North, Northeast, Southeast, South, and Center-West). For each region, we obtained the distribution of mean food intakes, excluding strata in which the food of interest had not been reported. We calculated the 10th percentile (lower limit) and 70th, 80th and 90th percentiles, which represented three levels of acceptability constraints: rigorous, moderate, and flexible (hereafter these are referred to as “acceptability levels”). This acceptability terminology reflects flexibility in the modification of food quantities relative to the usual food patterns allowed in optimized models. That is, rigorous constraints allowed a smaller difference from the usual intake than did moderate constraints, which in turn allowed a smaller difference than flexible constraints. The acceptability constraints imposed in a given GS were those obtained for its corresponding region.

**Fig. 1:**
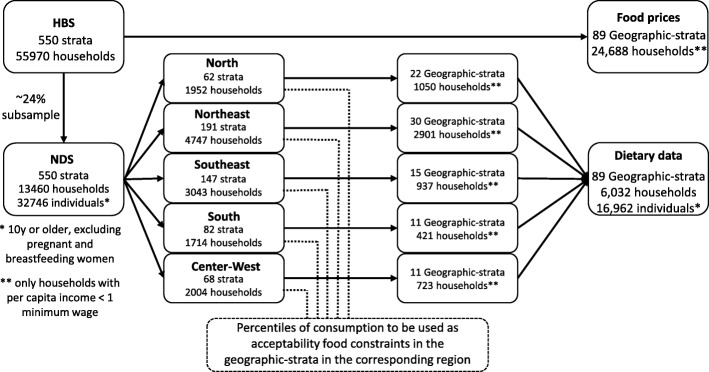
Description of the samples used to obtain food prices and consumption, and the acceptability constraints

These boundaries were obtained considering all samples and not only low-income households, to remove the potential effect of the income constraint in the food patterns among low-income households. For example, low-income individuals who eat few dairy products might not eat more because they cannot afford dairy rather than because dairy is unacceptable to them. Our assumption was that low-income people would eat more expensive foods as much as higher-income people living in the same region eat. Figure 1 presents a flowchart that describes the samples used to obtain food prices, consumption, and acceptability constraints.

### Objective functions

One objective function was used at each step. In the first step, the objective function minimized the relative difference between the observed and the optimized food quantities (eq. 1), while all nutritional constraints were imposed. In the second step, all nutritional constraints unfeasible in step 1 were removed. Then an additional factor, including undesirable deviations for limiting nutrients, was included in the objective function (eq. 2). Undesirable deviation refers to the difference between the target and optimized content of a limiting nutrient [26]. For example, for an essential nutrient with a target of 100 mg, an undesirable negative deviation of 10 mg refers to an optimized diet having only 90 mg of the nutrient instead 100 mg. Similarly, for harmful components such as trans-fat, for a target of 2 g an undesirable positive deviation of 0.5 g refers to an optimized diet having 2.5 g instead. The deviation for a given nutrient represents the least optimized difference between the target and solution if the constraint cannot be attained.

A standardized deviation factor, that is, the proportional difference between the desired and actual nutrient content in relation to the target, was included in the optimization model. The factor was applied to all components found to cause model unfeasibility in a given GS, as described in the previous section. Models are described as follows:

$$ \mathit{\operatorname{Minimize}}\ Y={\sum}_{i=1}^{i=g}\left|\frac{Q_i^{opt}-{Q}_i^{obs}}{Q_i^{obs}}\right|\kern0.75em \left( step\ 1\right) $$

$$ \mathit{\operatorname{Minimize}}\ Y={\sum}_{i=1}^{i=g}\left|\frac{Q_i^{opt}-{Q}_i^{obs}}{Q_i^{obs}}\right|+{\sum}_{n=1}^{n=N}\left|\frac{nut_n^{opt}-{target}_n}{target_n}\right|\kern1.25em \left( step\ 2\right) $$

In these models, *Y* represents the objective function to be minimized, ${Q}_i^{opt}$ is the quantity of the food item *i* in the optimized diet, *g* is the total number of food items, ${Q}_i^{obs}$ is the mean quantity of *i* in the observed diet, and ${nut}_n^{opt}$ is the amount of nutrients *n* in the optimized diet (N = number of limiting nutrients identified in step 1.) This is a non-linear function due to the use of absolute function, which was then linearized to include a set of linear constraints, following a similar procedure to that described in Darmon et al. (2002) [9].

Results were presented for the observed and optimized diets as means and standard deviations for the food and nutrient quantities across the 89 GS, for each acceptability level. The distribution of changes (in %) of the optimized nutrient contents, in relation to the observed contents, for each nutrient and each GS were plotted in a box-plot graph. Additionally, we plotted the percentage of GS in which each component was attained in the observed and optimized diets. Both graphs refer to the cost-constrained models.

## Results

General characteristics of the sample are described in Table 2. In the first step of the analysis, there was no feasible solution for any GS. That is, at least one limiting nutrient occurred in every GS. The most frequent limiting nutrients were calcium; vitamins D, E, and A; zinc; fiber; sodium; and saturated and trans-fat.

**Table 2:** General characteristic of people in low-income households^a^ in Brazil, 2008–2009

Variables	Percent
Sex	
*Male*	*45.8*
Age (years)	
*10–19*	*29.5*
*20–59*	*62.7*
*60 or more*	*7.8*
Years of schooling	
*< 4*	*33.1*
*4–8*	*36.2*
*8 or more*	*30.7*
	median (interquartile range)
Household members	2 (2)
Household per capita income (US$/month) ^b^	229.8 (166.4)

^a^ restricted to individuals with dietary data (*n* = 16,962)

In the second step, a deviance term was included to relax the exigence for the limiting nutrients identified in step 1 for each GS. Table 3 presents the nutrient contents in the mean observed and optimized diets, according to the acceptability level and cost constraint. Compared with the mean observed diet, the cost-constrained modeled diets presented the following results: i) higher amounts of fiber and most vitamins and minerals; and ii) similar amounts of macronutrients, fats, free sugar, and sodium. The most important increases were observed for fiber (up to 46%), calcium (up to 42%), copper (up to 51%), magnesium (up to 33%), vitamin A (up to 64%), vitamin C (up to 138%), and vitamin E (up to 28%). For most nutrients, the best achievement was obtained with cost-free models (Table 3).

**Table 3:** Mean (standard deviation) nutrient contents in the observed diets (i.e. mean observed diet) and in diets optimized with cost-constrained and cost-free models at three levels of acceptability constraints^a^ (*n* = 89 GS^b^)

Nutrient	Unit	Mean observed diet	Rigorous		Moderate		Flexible	
			cost constrained	cost free	cost constrained	cost free	cost constrained	cost free
Energy	kcal	1792 (226)	1792 (226)	1792 (226)	1792 (226)	1792 (226)	1792 (226)	1792 (226)
Carbohydrates	% kcal	51.7 (2)	51.3 (2.1)	51.6 (2.1)	50.6 (2.5)	51.4 (2.1)	50.4 (2.6)	52.6 (2.5)
Free sugars	%kcal	8.4 (2.7)	6.2 (2.2)	6.6 (2.2)	7.4 (2.4)	6.6 (2.2)	7 (2.4)	5.3 (1.5)
Total fiber	g	19.9 (3.2)	23.4 (2.4)	22.7 (2.4)	23.7 (3.1)	22.6 (2.8)	25.4 (3.5)	27.7 (3.2)
Protein	%kcal	18.3 (1.8)	19.7 (1.8)	19.3 (1.8)	20.5 (2.3)	19.3 (1.9)	21 (2.2)	19.4 (2)
Total fat	%kcal	30 (2.2)	30.4 (2.2)	30.2 (2.2)	30.5 (2.2)	30.4 (2.2)	30.6 (2.1)	30.3 (2)
Linoleic acid	%kcal	4.8 (0.4)	5.3 (0. 4)	5.1 (0.1)	5.1 (0.1)	5.1 (0.1)	5.3 (0.1)	5.6 (0.1)
Linolenic acid	%kcal	0.6 (0.1)	0.7 (0. 1)	0.7 (0.4)	0.7 (0.4)	0.6 (0.5)	0.7 (0.5)	0.7 (0.5)
Saturated fat	%kcal	10.4 (1.2)	10.0 (1.1)	10.1 (1.1)	10.2 (1.2)	10.2 (1.1)	10.0 (1.1)	9.9 (1)
Trans fat	%kcal	1.15 (0.3)	1.00 (0.2)	1.00 (0.2)	1.00 (0.2)	1.04 (0.2)	0.94 (0.2)	0.93 (0.1)
PUFA	%kcal	6.16 (0.4)	6.9 (0.5)	6.59 (0.5)	6.8 (0.4)	6.65 (0.5)	7.02 (0.5)	7.05 (0.5)
MUFA	kcal	10.03 (0.9)	10.0 (0.9)	10.0 (0.9)	10.0 (0.9)	10.0 (0.9)	10.0 (0.9)	10.0 (0.9)
Sodium	mg	2995 (352)	2962 (352)	2963 (329)	2963 (350)	2967 (331)	2894 (352)	2954 (340)
Calcium	mg	456 (75)	538 (78)	598 (77)	556 (72)	655 (94)	646 (94)	685 (111)
Copper	mg	1.32 (0.5)	1.75 (0.35)	1.71 (0.36)	1.74 (0.35)	1.76 (0.41)	1.99 (0.4)	1.9 (0.44)
Iron	mg	11.57 (1.7)	12.67 (1.24)	13.13 (1.35)	12.62 (1.26)	13.63 (1.38)	13.87 (1.15)	14.11 (1.38)
Phosphorus	mg	975 (161)	1049 (158)	1132 (159)	1056 (157)	1175 (167)	1109 (160)	1200 (202)
Magnesium	mg	228.9 (33)	262 (29.2)	281.6 (31)	261.5 (29.8)	298.9 (34.6)	305 (31)	328.2 (51.8)
Zinc	mg	10.9 (1.7)	11.2 (1.5)	11.3 (1.5)	11.1 (1.5)	11.4 (1.4)	11.3 (1.4)	11.5 (1.5)
Niacin	mg	25.1 (3.9)	27.8 (3.5)	29.9 (3.8)	27.9 (3.5)	31 (3.7)	28.7 (4.1)	31.2 (4.8)
Vitamin A ^c^	mcg	632 (342)	906 (247)	959 (240)	907 (242)	1019 (270)	1036 (276)	1062 (285)
Thiamin	mg	1.14 (0.2)	1.24 (0.17)	1.25 (0.17)	1.23 (0.17)	1.28 (0.17)	1.28 (0.17)	1.28 (0.17)
Riboflavin	mg	1.37 (0.3)	1.51 (0.25)	1.52 (0.23)	1.51 (0.25)	1.53 (0.23)	1.55 (0.23)	1.57 (0.22)
Vitamin B6	mg	1.43 (0.2)	1.52 (0.17)	1.62 (0.17)	1.51 (0.17)	1.66 (0.17)	1.56 (0.19)	1.68 (0.26)
Vitamin B12	mcg	6.12 (3)	7.72 (2.31)	7.99 (2.66)	7.69 (2.36)	8.1 (3.1)	7.92 (2.82)	8.05 (2.66)
Vitamin C	mg	44.5 (17.4)	71.7 (19.2)	86.1 (21)	69.2 (19.5)	99 (20.4)	105.8 (27.5)	119.2 (35.1)
Vitamin D	mcg	3.32 (2.2)	3.55 (2.07)	4.34 (2.27)	3.55 (2.08)	4.59 (2.26)	3.57 (2.09)	4.59 (2.47)
Vitamin E	mg	5.47 (0.9)	5.92 (0.7)	6.74 (0.71)	5.92 (0.69)	7.28 (0.83)	6.99 (0.77)	7.69 (1.39)
Folate ^d^	mcg	416 (87)	447 (77)	461 (81)	446 (76)	468 (78)	465 (73)	472 (74)

^a^ The acceptability constraints reflect the flexibility in the modifications allowed in the optimized models from the usual food patterns. The rigorous constraints allow a smaller deviation from the usual intake than the moderate one, and this, a smaller than the flexible one

^b^ The unit of analysis were the 89 geographic-strata; which comprise 16,962 individuals

^c^ Micrograms of Retinol Equivalent Activity

^d^ Micrograms of Dietary Folate Equivalent

**Fig. 2:**
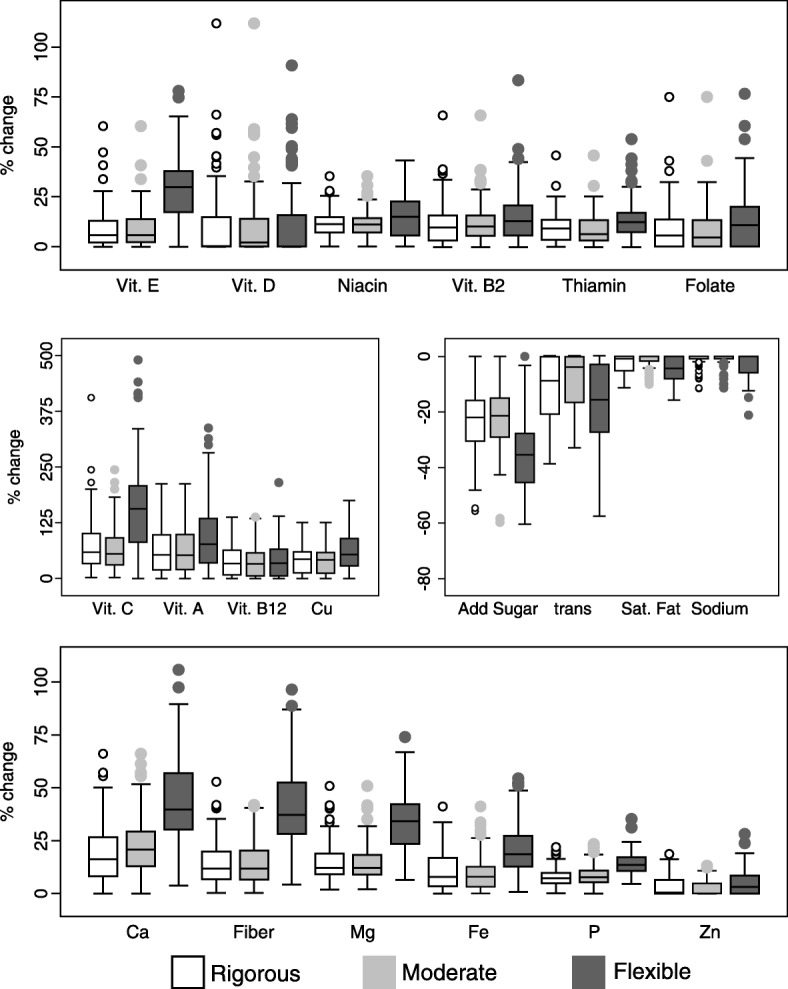
Box-plot of the distribution of the change in the nutrient contents for the cost-constrained models, according to the acceptability level, over the 89 GS related to the observed contents ^a^. ^a^ y-axis represents the deviance, in percentage, from the observed value

Figure 2 shows the distribution of the increase in nutrient contents for the cost-constrained models, according to the acceptability level, over the 89 GS, related to the observed contents; the y-axis represents deviance, as a percentage, from the observed values. Nutrient content increased in most GS, with high median increases for vitamins C and A, copper, calcium, magnesium, and fiber. The greatest reductions were observed for trans-fat and free sugars.

**Fig. 3:**
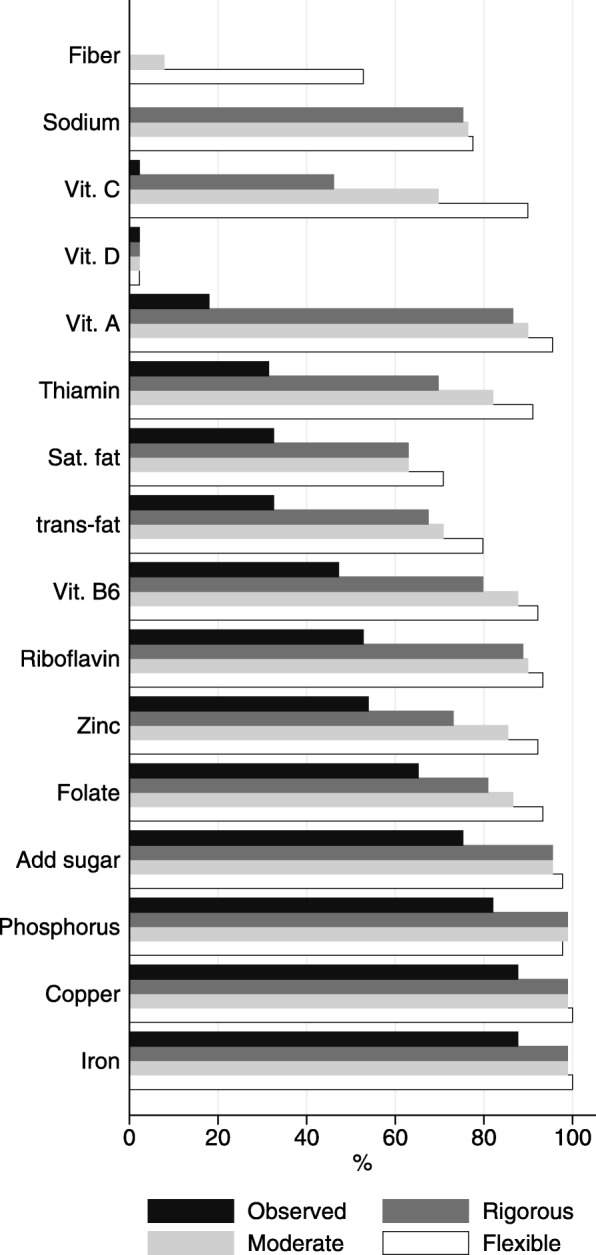
Percentage of GS meeting the recommendation for each nutrient in the observed and cost-constrained optimized diets, at three levels of acceptability constraints ^a^. ^a^ bars for calcium, magnesium, and vitamin E were omitted in the graph because the percentages were 0%, and for vit. B12 (percentages were 100%) in both observed and optimized diets

However, the increase in nutrient contents was not enough to meet the nutrient requirements in all GS. Figure 3 shows the percentage of GS in which the constraint was met for each nutrient in the observed and cost-constrained optimized diets. The optimized diets provided adequate amounts of most nutrients in at least 80% of the GS. The components for which it was challenging to satisfy the constraint were fiber, vitamins D and E, calcium, and magnesium. (The graph does not show bars for calcium, magnesium, and vitamin E because the percentages were 0% in both the observed and optimized diets).

Concerning food quantities in the optimized models, the most noticeable results were as follows. Fruits and beans increased in all models, but increases were greater in the cost-free and/or flexible models; large increase in whole cereals was observed only in the flexible models; large increase in vegetables (other than leafy vegetables) was observed only in the cost-free models; and fish increased only in the cost-free models (Table 4). For most items – such as fruits, beans, fish and seafood, yogurt, and non-fat milk – the increase was more important in the flexible model. Among items with reduced amounts in the optimized diets, the greatest reductions were noted for rice (mainly in cost-free models), red meat (mainly cost-constrained models), processed meats, sugar-sweetened beverages, and sweets. In general, diets optimized with cost-free models presented higher amounts of low-energy-density foods such as fruits, vegetables and legumes, and fish and seafoods. Dairy content was not influenced by the cost constraint.

**Table 4:** Mean (standard deviation) food contents in the observed diets (i.e. mean observed diet) and in diets optimized with cost-constrained and cost-free models at three levels of acceptability constraints^a^ (n = 89 GS^b^)

Foods / food groups (grams/day)	Observed diet	Rigorous		Moderate		Flexible	
		Cost constrained	Cost free	Cost constrained	Cost free	Cost constrained	Cost free
FV, legumes, seeds							
Leafy vegetables	3.3 (4.2)	7.5 (6.9)	8.1 (7.6)	7.3 (7.0)	9.6 (8.5)	12.1 (11.8)	13.9 (13.1)
Other vegetables	112.9 (77.7)	117.8 (73.1)	206.8 (85.1)	116.9 (71.2)	252.2 (103.3)	138.1 (80.7)	213.4 (120.1)
Tuber	22.3 (15.7)	34.3 (11.7)	34.0 (11.8)	34.0 (11.7)	37.2 (14.4)	38.2 (23.9)	39.8 (22.8)
Fruits	69.1 (30.0)	105.2 (29.3)	122.3 (33.7)	102.1 (29)	142.1 (35.8)	172.5 (58.8)	171.7 (55.7)
Beans	183.4 (71.9)	224.5 (40.2)	223.7 (42.8)	224.3 (40.3)	242.1 (43)	291.4 (30.4)	290.7 (34.3)
Nuts	0.1 (0.3)	0.6 (0.4)	0.6 (0.5)	0.5 (0.4)	0.7 (0.6)	2.5 (1.8)	2.1 (1.7)
Cereals							
Rice	161.2 (55.6)	117 (46.4)	94.3 (31.9)	114.2 (48.1)	84.1 (15.4)	92.6 (28.5)	86.4 (19.7)
Whole cereals	8.4 (12.7)	13.5 (13)	9.8 (11.3)	13.6 (12.9)	11.4 (14.3)	29.1 (26.6)	30.7 (26.5)
Pasta	43.4 (22.2)	53.5 (17.3)	48.8 (17.1)	53.5 (16.9)	43.5 (20.9)	60.1 (25.2)	48.4 (28.9)
Cake, cookies	27.1 (10.4)	28.8 (10.6)	27.7 (10.7)	26.5 (12.1)	25.8 (13.3)	16.8 (10.2)	18.8 (11.9)
Breads	53.4 (24.4)	44.4 (20.5)	35.7 (20.1)	43.2 (21.3)	27.3 (17.7)	27.2 (16.7)	23.7 (13.0)
Dairy							
Cheese	3.3 (3.2)	3.8 (3.6)	4.2 (3.5)	4.4 (3.4)	3.7 (4.2)	3.2 (4.3)	3.1 (4.3)
Yogurt	5.8 (6.2)	9.1 (7.0)	11.8 (7.4)	10.9 (7.5)	13.5 (8.2)	18.0 (13.5)	16.2 (12.6)
Non-fat milk	4.4 (7.5)	9.3 (7.9)	9.1 (7.9)	9.3 (7.9)	11.4 (8.6)	16.5 (12.6)	17.8 (12.6)
Milk	74.2 (40.9)	99.7 (33.4)	98.5 (32.0)	104.6 (31.2)	113.1 (38.4)	138.9 (52.6)	149.5 (45.9)
Meats, eggs							
Red meat	78.5 (24.4)	75.3 (24.7)	71.2 (17.7)	69.8 (21.5)	68.7 (16.9)	65.3 (14.9)	65.3 (14.3)
Chicken	39.4 (13.2)	38.6 (10.8)	40.8 (12.8)	44.8 (10.4)	39.6 (13.9)	48.9 (20)	46.7 (18.7)
Processed meat	5.7 (4.5)	6.1 (4.3)	4.3 (4.6)	5.9 (4.4)	4.0 (4.8)	7.6 (5.9)	5.9 (5.7)
Eggs	14.5 (6.9)	16.8 (5.8)	16.9 (5.6)	16.9 (5.6)	19.2 (5.0)	22.3 (6.2)	21.7 (6.7)
Fish, seafoods	45.5 (62.1)	44.8 (58.6)	66.9 (64.0)	44.5 (59.1)	69.8 (63.6)	36.1 (58.8)	61.5 (69.6)
Oils							
Margarine, butter	6.0 (3.0)	3.8 (2.5)	3.8 (2.7)	4.3 (2.7)	2.9 (2.1)	3.3 (1.9)	3 (2.2)
Olive	0.02 (0.1)	0.1 (0.1)	0.1 (0.1)	0.1 (0.1)	0.2 (0.1)	0.3 (0.1)	0.3 (0.1)
Other							
SSB ^c^	98.7 (56.0)	54.3 (37.1)	87.0 (60.1)	51.8 (34.3)	88.0 (67.1)	41.0 (16.3)	47.1 (32.1)
Snacks	51.1 (36.0)	68.5 (38.3)	71.7 (35.7)	68.7 (38.8)	77.2 (35.4)	58.2 (44.3)	59.1 (43.4)
Sweets	41.7 (39.4)	24.5 (33.1)	26.8 (32.6)	25.2 (33.1)	20.4 (28.1)	14.2 (18.5)	15.3 (20.0)
Manioc flour	16.5 (26.9)	22.0 (27.1)	16.5 (22.7)	21.6 (26.9)	15.0 (20.8)	20.9 (22.4)	15.2 (18.8)
Mean observed cost (US$)	2.16 (0.37)	2.16 (0.37)	2.70 (0.41)	2.16 (0.37)	2.90 (0.48)	2.16 (0.37)	2.60 (0.69)

^a^ The acceptability constraints reflect the flexibility in the modifications allowed in the optimized models from the usual food patterns. The rigorous constraints allow a smaller deviation from the usual intake than the moderate one, and this, a smaller than the flexible one

^b^ The unit of analysis were the 89 geographic-strata; which comprise 16,962 individuals

^c^ Sugar-sweetened beverage

The mean observed cost was R$4.98 (US$2.16^1^) per person/day. Diet cost increased in all cost-free models. The mean increase was greater in the moderate model (R$6.70; US$2.90) than in the rigorous (R$6.24; US$2.70) or flexible (R$5.99; US$2.60) models.

## Discussion

Using linear programming, we demonstrated that improving dietary quality is possible without increasing the current diet cost in low-income Brazilian households. However, diets in the cost-free models were consistent with the best nutrient content. Dietary changes identified were within the usual variability ranges in current food patterns. Few substantial changes were noted and only for certain food items. Thus, optimized diets accounted for two important barriers to the adoption of healthier choices, namely social acceptability and affordability, which stands in favor to consider these modifications as realistic and feasible to be adopted by individuals in low-income households.

The implications of the results are as follows. First, it is possible to increase the nutrient content of diets by changing only food choices, within a realistic range for quantity. The more flexible the acceptability constraints (i.e., the higher the departure allowed from the observed patterns), the higher the quantity of beneficial nutrients that can be provided by an optimized diet. Second, diet cost is indeed a barrier to increasing the nutrient content of diets. Diets optimized with cost-free models were more expensive financially than the mean observed diet; they also had better nutrient content than cost-constrained optimized diets. However, even with cost-free models and flexible acceptability constraints, it was not mathematically possible to strictly meet all nutrient recommendations in the optimized diets. In this case, the impact of hypothetical food affordability would lead to an increase in nutrient content; however, because of people’s food preferences, full adequacy could not be guaranteed. The acceptability boundaries themselves might be determined by food prices. That is, it might happen that if the prices were lower than actual prices, people would consume more of certain foods, which in turn would reflect in a higher preference, and hence higher acceptability boundaries. Indeed, this is expected to happen. In fact, in a study using regression analysis to estimate elasticity coefficients, the participation of fruits and vegetables in Brazilian household’s food basket would increase by 0.2% for each 1% decrease in price; the coefficients were significant for all income strata [7].

For certain nutrients it was highly challenging to meet the requirements, regardless of cost and acceptability level. That was the case for calcium, magnesium, vitamins D and E, fiber, and saturated and trans-fats. These nutrients are described as inadequate in both low- and high-income families in Brazil as well as in developed countries [1, 2]. However, optimized diets in developed countries have found solutions that cover a wide range of nutrient-based recommendations. Maillot et al. (2017) [19] designed nutritionally adequate diets for each individual in a representative sample of French adults, without increasing the cost, irrespective of the initial observed cost. Compared with the observed diets, optimization increased all plant-based foods, and decreased almost all animal-based foods except milk and fish. Also, in the absence of a cost constraint, dietary cost increased by 0.22 euros/d on average to reach nutritional adequacy. In another recent study, Parlesak et al. (2016) [27] modelled diets that allowed greater food variety. Their models achieved all recommended intakes with exception for vitamin D. In Brazil, there is so far only one published study using optimization to find the best diet modifications. Using a cost-free model and modeling the mean population food intakes, the study failed to reach the target for vitamin D, vitamin E, calcium, zinc, linolenic acid, and mono-unsaturated fat [28].

In the present study, diets optimized with cost-free models presented higher nutrient contents, especially concerning vitamins C, D, and E, compared with diets optimized using cost-constrained models. Among cost-free models, the best nutrient content was obtained with the flexible model. The resulting diet was associated with larger deviance from the mean observed diet, but also with a lower total cost compared with diets obtained from the rigorous and moderate models (US$2.60 vs. US$2.70 and US$2.90 respectively). This is in line with a previous study in the French context that showed that modest changes in habitual diet, mainly in the form of an increase in fruit and vegetables, were required to achieve nutrient recommendations in cost-free models. However, when a cost constraint was included and strengthened, departure from the average French diet progressively increased in the optimized diets [29]. In another study, nutritionally adequate modeled diets had their cost directly minimized; the results showed that cost increased both with the level of nutritional exigency and with the level of acceptability constraints [30]. Nonetheless, the impact of the cost constraint on food quantities in the present study was restricted to a few foods. These included all vegetables except leafy ones, fish and seafoods, and fruits. For other food items, with few exceptions, the quantities presented only small variations across all the models.

The limiting nutrients were calcium, magnesium, vitamins D and E, and fiber. Their main sources are fish, dairy products, fruits and vegetables, and vegetable oils [31]. Hence, higher contents of these foods in optimized diets would be necessary to reduce the inadequacies. However, important increases in these food groups were not tolerated in the optimized diets because acceptability constraints introduced in the models. Vitamin D was one of the limiting nutrients, however its requirement assumes minimal sun exposition [23] and might not be directly applied for the Brazilian population due to the relatively high exposure to sunlight in tropical countries [31]. A previous study in Brazil that measured 25(OH) D serum throughout the four seasons, indicated a mean of 50 mmol/L, which means that approximately 50% of the sample presented vitamin D deficiency, although 99% had an inadequate dietary vitamin D intake [32]. Concerning vitamin E, the WHO/FAO report stated that there is insufficient information to define indicators for vitamin E adequacy [31]. Existing values are based on the mean intake observed in the US and European countries, with vitamin E deficiency rarely being reported in humans. Given the evidence, it might be that the issue on nutrient inadequacy in the Brazilian population should be focused on calcium, magnesium, and fiber.

The results of the present study were obtained from a nationally representative individual-based dataset referring to food intake and prices collected during a 1-year period. Monthly variations in price, purchasing and consumption due to seasonality were thus accounted for. Additionally, our innovative geographic stratum (GS) modelling approach accounted for dietary habits and prices among small and predefined areas. Furthermore, the Brazilian HBS and NDS provide a unique opportunity to combine food consumption information and prices for the same households, referring to the same week of data collection. It provides the advantage of capturing both food price and consumption variations across the country, which are due to cultural and economic factors.

An important limitation of the study was the chosen set of nutrient intake recommendations. The RDAs were established for the US population, considering local anthropometric and food consumption information. Another limitation concerns the use of the food composition table based on USDA information. To date, Brazilian food composition tables remain rather limited; the data on food composition are insufficient to analyze a wide variety of food items and nutrients within national surveys. Nonetheless, food composition datasets from USDA have been used to estimate the prevalence of nutrient intake inadequacy in Brazilian population-based surveys [3, 4]. In addition, both the current observed diets and the optimized diets obtained through linear programming were subject to the same uncertainties regarding nutritional recommendations and food composition data.

The expected underreporting of true dietary intake probably resulted in underestimations of the mean observed intake and cost. Thus, the difference, in grams, of food contents in the optimized diets from the current observed intakes are probably overestimated. Finally, the data we analyzed were collected 10 years ago – yet provide the most recent nationwide data on food consumption and price in Brazil. Changes in food consumption patterns have likely occurred, as well as in prices and income, in the last decade. The extent of this limitation can be quantified only when the next HBS is conducted.

## Conclusions

This study showed that feasible changes are possible and would be compatible with existing dietary patterns among low-income individuals in Brazil. Such changes would substantially improve diet quality by improving nutrient content, without additional costs. However, increasing food budget would help to improve nutritional quality. Either way, complete nutritional adequacy seems unattainable, even when costs are not constrained and the acceptability level is not stringent. These results suggest that more dramatic dietary changes may be required.

## Data Availability

The datasets analyzed during the current study are available in the Brazilian Institute of Geography and Statistics’ repository (in Portuguese), [https://www.ibge.gov.br/estatisticas/sociais/saude/9050-pesquisa-de-orcamentos-familiares.html?edicao=9051&t=microdados].

## Abbreviations

BRL: Brazilian Reals
DRI: Dietary Reference Intakes
HBS: Household Budget Survey
NDS: National Dietary Survey
RDA: Recommended Dietary Allowances
US$: United States Dollars
USDA: United States Department of Agriculture
WHO/FAO: World Health Organization / Food and Agriculture Organization

## Footnotes

1 US$1.00 = R$2.31 (January 30, 2009).
